# 3-Hy­droxy-4-phenyl-1-(prop-2-en-1-yl)-2,3,4,5-tetra­hydro-1*H*-1,5-benzodiazepin-2-one

**DOI:** 10.1107/S1600536811054456

**Published:** 2011-12-23

**Authors:** Mohamed Rida, Khalil Mamari, El Mokhtar Essassi, Seik Weng Ng

**Affiliations:** a Laboratoire de Chimie Organique Hétérocyclique, Pôle de Compétences Pharmacochimie, Université Mohammed V-Agdal, BP 1014 Avenue Ibn Batout, Rabat, Morocco; b Department of Chemistry, University of Malaya, 50603 Kuala Lumpur, Malaysia; c Chemistry Department, King Abdulaziz University, PO Box 80203 Jeddah, Saudi Arabia

## Abstract

The asymmetric unit of the title compound, C_18_H_18_N_2_O_2_, contains three independent mol­ecules. In each, the seven-membered diazepine ring adopts a boat conformation with the hy­droxy-substituted C atom at the prow and fused-ring C atoms at the stern. In the crystal, the mol­ecules are linked by O—H⋯O and N—H⋯O hydrogen bonds. The allyl group of one mol­ecule is equally disordered over two positions.

## Related literature

For a related structure, see: Rida *et al.* (2011 ▶).
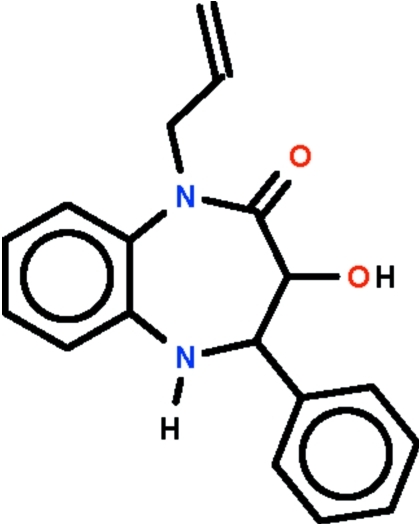


## Experimental

### 

#### Crystal data

C_18_H_18_N_2_O_2_
*M* *_r_* = 294.34
Monoclinic, 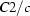

*a* = 51.6665 (8) Å
*b* = 14.5766 (2) Å
*c* = 11.9316 (2) Å
β = 90.965 (2)°
*V* = 8984.7 (2) Å^3^
*Z* = 24
Mo *K*α radiation
μ = 0.09 mm^−1^
*T* = 293 K
0.21 × 0.15 × 0.13 mm

#### Data collection

Bruker APEX DUO diffractometer
98635 measured reflections
11182 independent reflections
8815 reflections with *I* > 2σ(*I*)
*R*_int_ = 0.045

#### Refinement

*R*[*F* ^2^ > 2σ(*F* ^2^)] = 0.052
*wR*(*F* ^2^) = 0.153
*S* = 1.01
11182 reflections
604 parameters
16 restraints
H-atom parameters constrained
Δρ_max_ = 0.72 e Å^−3^
Δρ_min_ = −0.74 e Å^−3^

### 

Data collection: *APEX2* (Bruker, 2010 ▶); cell refinement: *SAINT* (Bruker, 2010 ▶); data reduction: *SAINT*; program(s) used to solve structure: *SHELXS97* (Sheldrick, 2008 ▶); program(s) used to refine structure: *SHELXL97* (Sheldrick, 2008 ▶); molecular graphics: *X-SEED* (Barbour, 2001 ▶); software used to prepare material for publication: *publCIF* (Westrip, 2010 ▶).

## Supplementary Material

Crystal structure: contains datablock(s) global, I. DOI: 10.1107/S1600536811054456/xu5411sup1.cif

Structure factors: contains datablock(s) I. DOI: 10.1107/S1600536811054456/xu5411Isup2.hkl

Supplementary material file. DOI: 10.1107/S1600536811054456/xu5411Isup3.cml

Additional supplementary materials:  crystallographic information; 3D view; checkCIF report

## Figures and Tables

**Table 1:** Hydrogen-bond geometry (Å, °)

*D*—H⋯*A*	*D*—H	H⋯*A*	*D*⋯*A*	*D*—H⋯*A*
O1—H1o⋯O2^i^	0.84	1.96	2.786 (2)	167
O5—H5o⋯O6^ii^	0.84	2.30	3.022 (2)	145
N1—H1n⋯O3	0.88	2.58	3.142 (2)	123
N3—H3n⋯O4^iii^	0.88	2.40	2.900 (2)	116

Symmetry codes: (i) 
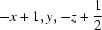
; (ii) 
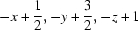
; (iii) 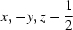.

**Fig. 1.**
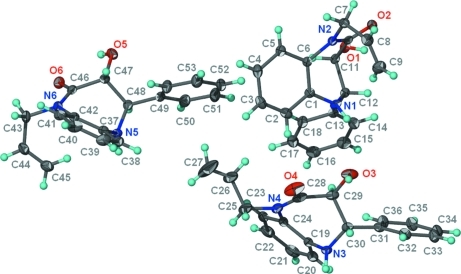
Thermal ellipsoid plot (Barbour, 2001) of the three independent molecules of C18H18N2O2 at the 50% probability level; hydrogen atoms are drawn as spheres of arbitrary radius. The disorder in one allyl group is not shown.

## supplementary crystallographic information

### Comment

A previous study describes 3-hydroxy-4-phenyl-1-[(3-phenyl-4,5-dihydro-1,2-oxazol-5-yl)methyl]-4,5-dihydro-1*H*-1,5-benzodiazepine-2(3*H*)-one (Rida *et al.*, 2011). The present study has an ally group in place of the 3-phenyl-4,5-dihyro-1,2-oxazol-5-yl group. The compound, C_18_H_18_N_2_O_2_ (Scheme I) crystallizes as three independent molecules, one of which has the allyl group disordered over two positions (Fig. 1). In these three molecules, the seven-membered diazepine ring adopts a boat conformation with the hydroxy-substituted C atom at the prow and fused-ring C atoms at the stern.

Despite the present of amino and hydroxy groups, the crystal structure features few hydrogen bonds (Table 1).

### Experimental

To a solution of 3-hydroxy-4-phenyl-4,5-dihydro-1*H*-1,5-benzodiazepin-2(3*H*)-one (1 g, 3.5 mmol) in DMF (20 ml) were added allyl bromide (0.5 g. 4.2 mmol), potassium carbonate (1 g, 7.4 mmol) and a catalytic quantity of tetra-*n*-buthyl ammonium bromide. The mixture was stirred at room temperature for 24 hours. The solution was filtered and the solvent removed under reduced pressure. The residue was recrystallized from ethanol to afford the compound as colorless crystals.

### Refinement

H-atoms were placed in calculated positions (C–H 0.93–0.98 and N–H 0.88 Å) and were included in the refinement in the riding model approximation, with *U*(H) set to 1.2*U*(C,N).

The allyl group of one molecule is disordered over two positions; the CH_2_–CH and C═C distances were tightly restrained to 1.50±0.005 and 1.40±0.005 Å. The temperature factors of the primed atom were set to those of the unprimed atom, and the anisotropic temperature factors were restrained to be nearly isotropic.

Omitted were (1 1 0), (4 0 0), (3 1 0) and (2 0 0).

### Crystal data

**Table:**

C_18_H_18_N_2_O_2_	*F*(000) = 3744
*M**_r_* = 294.34	*D*_x_ = 1.306 Mg m^−^^3^
Monoclinic, *C*2/*c*	Mo *K*α radiation, λ = 0.71073 Å
Hall symbol: -C 2yc	Cell parameters from 9776 reflections
*a* = 51.6665 (8) Å	θ = 2.5–27.5°
*b* = 14.5766 (2) Å	µ = 0.09 mm^−^^1^
*c* = 11.9316 (2) Å	*T* = 293 K
β = 90.965 (2)°	Prism, colorless
*V* = 8984.7 (2) Å^3^	0.21 × 0.15 × 0.13 mm
*Z* = 24	

### Data collection

**Table:**

Bruker APEX DUO diffractometer	8815 reflections with *I* > 2σ(*I*)
Radiation source: fine-focus sealed tube	*R*_int_ = 0.045
graphite	θ_max_ = 28.3°, θ_min_ = 2.3°
ω scans	*h* = −68→68
98635 measured reflections	*k* = −19→19
11182 independent reflections	*l* = −15→15

### Refinement

**Table:**

Refinement on *F*^2^	Primary atom site location: structure-invariant direct methods
Least-squares matrix: full	Secondary atom site location: difference Fourier map
*R*[*F*^2^ > 2σ(*F*^2^)] = 0.052	Hydrogen site location: inferred from neighbouring sites
*wR*(*F*^2^) = 0.153	H-atom parameters constrained
*S* = 1.01	*w* = 1/[σ^2^(*F*_o_^2^) + (0.0786*P*)^2^ + 10.8059*P*] where *P* = (*F*_o_^2^ + 2*F*_c_^2^)/3
11182 reflections	(Δ/σ)_max_ = 0.001
604 parameters	Δρ_max_ = 0.72 e Å^−^^3^
16 restraints	Δρ_min_ = −0.74 e Å^−^^3^

### Fractional atomic coordinates and isotropic or equivalent isotropic displacement parameters (Å^2^)

**Table:**

	*x*	*y*	*z*	*U*_iso_*/*U*_eq_	Occ. (<1)
O1	0.46395 (2)	0.49919 (8)	0.24790 (9)	0.0258 (2)	
H1O	0.4768	0.4833	0.2104	0.039*	
O2	0.49909 (2)	0.42796 (8)	0.39543 (9)	0.0254 (2)	
O3	0.42884 (3)	0.12182 (9)	0.12871 (12)	0.0417 (3)	
H3O	0.4289	0.0757	0.1710	0.063*	
O4	0.38520 (4)	0.08749 (10)	0.23349 (11)	0.0558 (5)	
O5	0.28615 (2)	0.65848 (8)	0.53439 (11)	0.0317 (3)	
H5O	0.2754	0.6950	0.5054	0.048*	
O6	0.23456 (2)	0.65290 (8)	0.52216 (10)	0.0295 (3)	
N1	0.44510 (3)	0.26012 (9)	0.31924 (11)	0.0240 (3)	
H1N	0.4495	0.2041	0.2995	0.029*	
N2	0.46785 (2)	0.36338 (9)	0.50005 (10)	0.0199 (3)	
N3	0.38017 (3)	0.03270 (9)	−0.07443 (11)	0.0245 (3)	
H3N	0.3738	−0.0220	−0.0895	0.029*	
N4	0.35925 (3)	0.14387 (11)	0.09582 (13)	0.0352 (4)	
N5	0.26551 (3)	0.42490 (10)	0.45243 (11)	0.0258 (3)	
H5N	0.2590	0.4013	0.3901	0.031*	
N6	0.23191 (3)	0.51238 (9)	0.60356 (11)	0.0227 (3)	
C1	0.43185 (3)	0.27309 (10)	0.41930 (13)	0.0215 (3)	
C2	0.40848 (3)	0.22715 (12)	0.43807 (14)	0.0280 (3)	
H2	0.4014	0.1900	0.3820	0.034*	
C3	0.39572 (3)	0.23611 (13)	0.53835 (15)	0.0313 (4)	
H3	0.3806	0.2031	0.5501	0.038*	
C4	0.40526 (3)	0.29392 (12)	0.62158 (14)	0.0289 (4)	
H4	0.3962	0.3021	0.6875	0.035*	
C5	0.42850 (3)	0.33939 (11)	0.60546 (13)	0.0244 (3)	
H5	0.4349	0.3790	0.6604	0.029*	
C6	0.44231 (3)	0.32611 (10)	0.50729 (12)	0.0203 (3)	
C7	0.48666 (3)	0.34345 (11)	0.59069 (13)	0.0238 (3)	
H7A	0.4777	0.3433	0.6614	0.029*	
H7B	0.4993	0.3925	0.5938	0.029*	
C8	0.50061 (3)	0.25390 (12)	0.57856 (15)	0.0310 (4)	
H8	0.5116	0.2371	0.6374	0.037*	
C9	0.49894 (4)	0.19689 (13)	0.49419 (19)	0.0407 (4)	
H9A	0.4882	0.2104	0.4331	0.049*	
H9B	0.5085	0.1429	0.4952	0.049*	
C10	0.47628 (3)	0.40596 (10)	0.40691 (12)	0.0199 (3)	
C11	0.45605 (3)	0.42556 (10)	0.31523 (12)	0.0208 (3)	
H11	0.4397	0.4419	0.3506	0.025*	
C12	0.45194 (3)	0.33759 (10)	0.24566 (12)	0.0209 (3)	
H12	0.4685	0.3226	0.2115	0.025*	
C13	0.43239 (3)	0.35188 (11)	0.15081 (13)	0.0225 (3)	
C14	0.44088 (3)	0.35804 (13)	0.04135 (14)	0.0293 (4)	
H14	0.4585	0.3560	0.0271	0.035*	
C15	0.42323 (4)	0.36731 (14)	−0.04743 (15)	0.0356 (4)	
H15	0.4291	0.3712	−0.1205	0.043*	
C16	0.39702 (4)	0.37079 (13)	−0.02703 (16)	0.0359 (4)	
H16	0.3852	0.3761	−0.0863	0.043*	
C17	0.38845 (4)	0.36631 (13)	0.08187 (17)	0.0350 (4)	
H17	0.3708	0.3698	0.0959	0.042*	
C18	0.40596 (3)	0.35664 (12)	0.17052 (15)	0.0288 (3)	
H18	0.4000	0.3533	0.2435	0.035*	
C19	0.36579 (3)	0.11140 (11)	−0.10302 (13)	0.0231 (3)	
C20	0.35985 (3)	0.13185 (12)	−0.21494 (15)	0.0292 (3)	
H20	0.3671	0.0970	−0.2715	0.035*	
C21	0.34330 (4)	0.20317 (14)	−0.24280 (19)	0.0396 (4)	
H21	0.3395	0.2159	−0.3177	0.048*	
C22	0.33236 (4)	0.25554 (14)	−0.1593 (2)	0.0464 (5)	
H22	0.3210	0.3029	−0.1780	0.056*	
C23	0.33826 (3)	0.23755 (13)	−0.0481 (2)	0.0403 (5)	
H23	0.3311	0.2735	0.0078	0.048*	
C24	0.35487 (3)	0.16635 (12)	−0.01918 (15)	0.0282 (3)	
C25	0.33697 (5)	0.14175 (17)	0.1726 (2)	0.0595 (8)	
H25A	0.3349	0.0800	0.2014	0.071*	0.50
H25B	0.3213	0.1575	0.1309	0.071*	0.50
H25C	0.3395	0.0930	0.2270	0.071*	0.50
H25D	0.3213	0.1284	0.1298	0.071*	0.50
C26	0.34037 (9)	0.2076 (3)	0.2700 (3)	0.0361 (8)	0.50
H26	0.3553	0.2106	0.3143	0.043*	0.50
C27	0.31929 (9)	0.2621 (3)	0.2860 (4)	0.0514 (9)	0.50
H27A	0.3048	0.2563	0.2392	0.062*	0.50
H27B	0.3194	0.3049	0.3437	0.062*	0.50
C26'	0.33372 (10)	0.2330 (3)	0.2343 (3)	0.0361 (8)	0.50
H26'	0.3338	0.2880	0.1949	0.043*	0.50
C27'	0.33081 (10)	0.2328 (3)	0.3450 (3)	0.0514 (9)	0.50
H27C	0.3308	0.1775	0.3840	0.062*	0.50
H27D	0.3288	0.2878	0.3833	0.062*	0.50
C28	0.38237 (4)	0.11630 (12)	0.13676 (14)	0.0336 (4)	
C29	0.40592 (3)	0.12239 (11)	0.06246 (13)	0.0272 (3)	
H29	0.4051	0.1797	0.0196	0.033*	
C30	0.40587 (3)	0.04118 (11)	−0.01933 (13)	0.0240 (3)	
H30	0.4087	−0.0145	0.0251	0.029*	
C31	0.42762 (3)	0.04703 (11)	−0.10277 (14)	0.0246 (3)	
C32	0.44697 (3)	−0.01911 (12)	−0.10342 (15)	0.0293 (4)	
H32	0.4472	−0.0651	−0.0494	0.035*	
C33	0.46606 (3)	−0.01722 (13)	−0.18410 (17)	0.0344 (4)	
H33	0.4789	−0.0618	−0.1836	0.041*	
C34	0.46599 (3)	0.05065 (13)	−0.26528 (16)	0.0327 (4)	
H34	0.4786	0.0514	−0.3198	0.039*	
C35	0.44694 (3)	0.11747 (13)	−0.26473 (16)	0.0330 (4)	
H35	0.4469	0.1635	−0.3187	0.040*	
C36	0.42802 (3)	0.11597 (12)	−0.18396 (16)	0.0310 (4)	
H36	0.4154	0.1615	−0.1838	0.037*	
C37	0.26079 (3)	0.38385 (11)	0.55772 (13)	0.0237 (3)	
C38	0.27134 (4)	0.29858 (12)	0.58668 (16)	0.0317 (4)	
H38	0.2825	0.2693	0.5377	0.038*	
C39	0.26540 (4)	0.25737 (12)	0.68735 (16)	0.0364 (4)	
H39	0.2724	0.2003	0.7053	0.044*	
C40	0.24913 (4)	0.30073 (13)	0.76138 (16)	0.0365 (4)	
H40	0.2452	0.2730	0.8292	0.044*	
C41	0.23863 (4)	0.38550 (12)	0.73449 (14)	0.0299 (4)	
H41	0.2279	0.4149	0.7849	0.036*	
C42	0.24410 (3)	0.42702 (11)	0.63247 (13)	0.0230 (3)	
C43	0.20376 (3)	0.52092 (12)	0.61385 (15)	0.0287 (3)	
H43A	0.1993	0.5855	0.6168	0.034*	
H43B	0.1986	0.4932	0.6840	0.034*	
C44	0.18876 (3)	0.47685 (12)	0.51980 (17)	0.0337 (4)	
H44	0.1708	0.4751	0.5259	0.040*	
C45	0.19871 (4)	0.44052 (13)	0.42947 (17)	0.0370 (4)	
H45A	0.2165	0.4408	0.4200	0.044*	
H45B	0.1879	0.4145	0.3750	0.044*	
C46	0.24510 (3)	0.58222 (10)	0.55556 (13)	0.0222 (3)	
C47	0.27447 (3)	0.57209 (11)	0.54834 (13)	0.0234 (3)	
H47	0.2810	0.5449	0.6184	0.028*	
C48	0.28166 (3)	0.50876 (11)	0.45054 (13)	0.0232 (3)	
H48	0.2774	0.5415	0.3809	0.028*	
C49	0.31030 (3)	0.48644 (11)	0.44854 (13)	0.0240 (3)	
C50	0.32412 (4)	0.49960 (12)	0.35145 (15)	0.0320 (4)	
H50	0.3159	0.5255	0.2891	0.038*	
C51	0.35011 (4)	0.47459 (14)	0.34579 (18)	0.0395 (4)	
H51	0.3591	0.4841	0.2800	0.047*	
C52	0.36235 (4)	0.43598 (13)	0.43717 (18)	0.0385 (4)	
H52	0.3797	0.4195	0.4336	0.046*	
C53	0.34883 (4)	0.42174 (15)	0.53443 (17)	0.0395 (4)	
H53	0.3571	0.3955	0.5964	0.047*	
C54	0.32298 (3)	0.44652 (14)	0.53984 (15)	0.0331 (4)	
H54	0.3140	0.4363	0.6056	0.040*	

### Atomic displacement parameters (Å^2^)

**Table:**

	*U*^11^	*U*^22^	*U*^33^	*U*^12^	*U*^13^	*U*^23^
O1	0.0316 (6)	0.0209 (5)	0.0252 (6)	0.0022 (4)	0.0077 (5)	0.0063 (4)
O2	0.0235 (6)	0.0288 (6)	0.0242 (5)	−0.0039 (4)	0.0041 (4)	−0.0014 (4)
O3	0.0538 (9)	0.0302 (7)	0.0402 (8)	0.0015 (6)	−0.0232 (6)	−0.0012 (6)
O4	0.1104 (15)	0.0351 (8)	0.0220 (6)	−0.0117 (8)	0.0094 (7)	0.0045 (6)
O5	0.0259 (6)	0.0239 (6)	0.0454 (7)	−0.0047 (5)	0.0033 (5)	0.0001 (5)
O6	0.0302 (6)	0.0221 (6)	0.0363 (7)	0.0039 (5)	0.0036 (5)	0.0023 (5)
N1	0.0324 (7)	0.0171 (6)	0.0226 (6)	0.0012 (5)	0.0008 (5)	−0.0016 (5)
N2	0.0211 (6)	0.0202 (6)	0.0183 (6)	−0.0008 (5)	0.0009 (5)	−0.0002 (5)
N3	0.0273 (7)	0.0195 (6)	0.0269 (7)	−0.0042 (5)	0.0016 (5)	−0.0043 (5)
N4	0.0412 (9)	0.0357 (8)	0.0292 (7)	−0.0154 (7)	0.0185 (7)	−0.0130 (6)
N5	0.0273 (7)	0.0282 (7)	0.0218 (6)	−0.0036 (6)	0.0010 (5)	−0.0066 (5)
N6	0.0213 (6)	0.0214 (6)	0.0256 (6)	0.0008 (5)	0.0032 (5)	−0.0004 (5)
C1	0.0247 (7)	0.0180 (7)	0.0218 (7)	0.0008 (6)	−0.0001 (6)	0.0026 (5)
C2	0.0277 (8)	0.0257 (8)	0.0305 (8)	−0.0044 (6)	−0.0040 (6)	0.0031 (6)
C3	0.0226 (8)	0.0330 (9)	0.0385 (9)	−0.0043 (7)	0.0021 (7)	0.0104 (7)
C4	0.0250 (8)	0.0350 (9)	0.0268 (8)	0.0035 (7)	0.0057 (6)	0.0082 (7)
C5	0.0258 (8)	0.0262 (8)	0.0214 (7)	0.0022 (6)	0.0021 (6)	0.0026 (6)
C6	0.0221 (7)	0.0180 (7)	0.0208 (7)	0.0004 (5)	0.0018 (5)	0.0031 (5)
C7	0.0252 (8)	0.0258 (8)	0.0204 (7)	−0.0011 (6)	−0.0014 (6)	0.0000 (6)
C8	0.0286 (8)	0.0298 (9)	0.0343 (9)	0.0037 (7)	−0.0043 (7)	0.0055 (7)
C9	0.0427 (11)	0.0267 (9)	0.0526 (12)	0.0100 (8)	−0.0050 (9)	−0.0047 (8)
C10	0.0242 (7)	0.0155 (6)	0.0200 (7)	0.0008 (5)	0.0037 (5)	−0.0032 (5)
C11	0.0239 (7)	0.0193 (7)	0.0195 (7)	0.0018 (6)	0.0042 (5)	0.0018 (5)
C12	0.0224 (7)	0.0218 (7)	0.0187 (7)	0.0015 (6)	0.0017 (5)	−0.0004 (5)
C13	0.0244 (8)	0.0213 (7)	0.0217 (7)	0.0008 (6)	−0.0006 (6)	0.0004 (6)
C14	0.0268 (8)	0.0375 (9)	0.0236 (8)	−0.0023 (7)	0.0008 (6)	0.0022 (7)
C15	0.0439 (11)	0.0387 (10)	0.0241 (8)	−0.0025 (8)	−0.0052 (7)	0.0033 (7)
C16	0.0407 (10)	0.0309 (9)	0.0355 (9)	0.0048 (8)	−0.0162 (8)	0.0007 (7)
C17	0.0256 (9)	0.0347 (9)	0.0447 (10)	0.0058 (7)	−0.0062 (7)	−0.0030 (8)
C18	0.0266 (8)	0.0314 (8)	0.0287 (8)	0.0043 (7)	0.0018 (6)	−0.0009 (7)
C19	0.0199 (7)	0.0219 (7)	0.0278 (8)	−0.0045 (6)	0.0044 (6)	−0.0018 (6)
C20	0.0263 (8)	0.0319 (9)	0.0296 (8)	−0.0050 (7)	0.0009 (6)	0.0005 (7)
C21	0.0321 (9)	0.0353 (10)	0.0509 (11)	−0.0080 (8)	−0.0137 (8)	0.0086 (9)
C22	0.0273 (9)	0.0282 (9)	0.0833 (16)	0.0007 (7)	−0.0142 (10)	−0.0039 (10)
C23	0.0212 (8)	0.0330 (9)	0.0668 (13)	−0.0017 (7)	0.0031 (8)	−0.0188 (9)
C24	0.0222 (8)	0.0266 (8)	0.0360 (9)	−0.0068 (6)	0.0081 (6)	−0.0084 (7)
C25	0.0658 (15)	0.0596 (14)	0.0545 (13)	−0.0373 (12)	0.0432 (12)	−0.0344 (11)
C26	0.041 (2)	0.045 (2)	0.0226 (19)	−0.0109 (15)	0.0109 (15)	−0.0086 (15)
C27	0.062 (2)	0.0432 (19)	0.049 (2)	−0.0150 (16)	0.0163 (16)	−0.0183 (15)
C26'	0.041 (2)	0.045 (2)	0.0226 (19)	−0.0109 (15)	0.0109 (15)	−0.0086 (15)
C27'	0.062 (2)	0.0432 (19)	0.049 (2)	−0.0150 (16)	0.0163 (16)	−0.0183 (15)
C28	0.0585 (12)	0.0208 (8)	0.0216 (8)	−0.0099 (8)	0.0070 (8)	−0.0029 (6)
C29	0.0382 (9)	0.0199 (7)	0.0232 (7)	−0.0017 (6)	−0.0059 (6)	0.0009 (6)
C30	0.0299 (8)	0.0183 (7)	0.0238 (7)	−0.0013 (6)	−0.0004 (6)	0.0018 (6)
C31	0.0254 (8)	0.0205 (7)	0.0276 (8)	−0.0027 (6)	−0.0023 (6)	0.0010 (6)
C32	0.0286 (8)	0.0229 (8)	0.0362 (9)	−0.0001 (6)	−0.0038 (7)	0.0068 (7)
C33	0.0243 (8)	0.0293 (9)	0.0496 (11)	0.0049 (7)	0.0006 (7)	0.0041 (8)
C34	0.0221 (8)	0.0348 (9)	0.0414 (10)	−0.0017 (7)	0.0042 (7)	0.0024 (8)
C35	0.0282 (9)	0.0318 (9)	0.0391 (10)	−0.0006 (7)	0.0023 (7)	0.0118 (7)
C36	0.0266 (8)	0.0272 (8)	0.0392 (9)	0.0048 (7)	0.0029 (7)	0.0088 (7)
C37	0.0232 (7)	0.0227 (7)	0.0253 (7)	−0.0018 (6)	−0.0011 (6)	−0.0028 (6)
C38	0.0313 (9)	0.0254 (8)	0.0383 (9)	0.0040 (7)	−0.0041 (7)	−0.0069 (7)
C39	0.0453 (11)	0.0216 (8)	0.0420 (10)	0.0039 (7)	−0.0111 (8)	0.0011 (7)
C40	0.0526 (12)	0.0275 (9)	0.0294 (9)	−0.0046 (8)	−0.0043 (8)	0.0058 (7)
C41	0.0370 (9)	0.0271 (8)	0.0255 (8)	−0.0036 (7)	0.0022 (7)	−0.0010 (6)
C42	0.0237 (7)	0.0206 (7)	0.0246 (7)	−0.0012 (6)	−0.0011 (6)	−0.0003 (6)
C43	0.0223 (8)	0.0298 (8)	0.0342 (9)	0.0025 (6)	0.0081 (6)	0.0003 (7)
C44	0.0204 (8)	0.0296 (9)	0.0512 (11)	0.0005 (7)	−0.0018 (7)	0.0015 (8)
C45	0.0311 (9)	0.0359 (10)	0.0436 (10)	−0.0020 (8)	−0.0097 (8)	−0.0043 (8)
C46	0.0253 (8)	0.0203 (7)	0.0211 (7)	0.0001 (6)	0.0022 (6)	−0.0033 (6)
C47	0.0240 (8)	0.0209 (7)	0.0254 (7)	−0.0018 (6)	0.0013 (6)	−0.0013 (6)
C48	0.0232 (7)	0.0258 (8)	0.0206 (7)	0.0009 (6)	0.0010 (6)	0.0002 (6)
C49	0.0236 (8)	0.0239 (7)	0.0247 (7)	0.0004 (6)	0.0029 (6)	−0.0038 (6)
C50	0.0355 (9)	0.0309 (9)	0.0298 (9)	0.0008 (7)	0.0079 (7)	0.0028 (7)
C51	0.0363 (10)	0.0356 (10)	0.0473 (11)	−0.0014 (8)	0.0195 (9)	−0.0003 (8)
C52	0.0232 (8)	0.0343 (10)	0.0583 (12)	0.0015 (7)	0.0067 (8)	−0.0105 (9)
C53	0.0317 (10)	0.0470 (11)	0.0398 (10)	0.0112 (8)	−0.0041 (8)	−0.0043 (9)
C54	0.0284 (9)	0.0464 (10)	0.0246 (8)	0.0064 (8)	0.0028 (6)	−0.0019 (7)

### Geometric parameters (Å, °)

**Table:**

O1—C11	1.4053 (18)	C22—H22	0.9300
O1—H1O	0.8400	C23—C24	1.386 (3)
O2—C10	1.2309 (19)	C23—H23	0.9300
O3—C29	1.413 (2)	C25—C26	1.515 (4)
O3—H3O	0.8400	C25—C26'	1.531 (4)
O4—C28	1.235 (2)	C25—H25A	0.9700
O5—C47	1.4073 (19)	C25—H25B	0.9700
O5—H5O	0.8400	C25—H25C	0.9700
O6—C46	1.229 (2)	C25—H25D	0.9700
N1—C1	1.399 (2)	C26—C27	1.364 (4)
N1—C12	1.477 (2)	C26—H26	0.9300
N1—H1N	0.8800	C27—H27A	0.9300
N2—C10	1.3513 (19)	C27—H27B	0.9300
N2—C6	1.4311 (19)	C26'—C27'	1.332 (4)
N2—C7	1.4706 (19)	C26'—H26'	0.9300
N3—C19	1.406 (2)	C27'—H27C	0.9300
N3—C30	1.477 (2)	C27'—H27D	0.9300
N3—H3N	0.8800	C28—C29	1.520 (3)
N4—C28	1.345 (3)	C29—C30	1.534 (2)
N4—C24	1.425 (2)	C29—H29	0.9800
N4—C25	1.484 (2)	C30—C31	1.516 (2)
N5—C37	1.416 (2)	C30—H30	0.9800
N5—C48	1.481 (2)	C31—C32	1.389 (2)
N5—H5N	0.8800	C31—C36	1.396 (2)
N6—C46	1.357 (2)	C32—C33	1.390 (3)
N6—C42	1.434 (2)	C32—H32	0.9300
N6—C43	1.467 (2)	C33—C34	1.384 (3)
C1—C2	1.402 (2)	C33—H33	0.9300
C1—C6	1.405 (2)	C34—C35	1.385 (3)
C2—C3	1.382 (2)	C34—H34	0.9300
C2—H2	0.9300	C35—C36	1.385 (3)
C3—C4	1.387 (3)	C35—H35	0.9300
C3—H3	0.9300	C36—H36	0.9300
C4—C5	1.388 (2)	C37—C38	1.398 (2)
C4—H4	0.9300	C37—C42	1.400 (2)
C5—C6	1.395 (2)	C38—C39	1.382 (3)
C5—H5	0.9300	C38—H38	0.9300
C7—C8	1.499 (2)	C39—C40	1.383 (3)
C7—H7A	0.9700	C39—H39	0.9300
C7—H7B	0.9700	C40—C41	1.385 (3)
C8—C9	1.307 (3)	C40—H40	0.9300
C8—H8	0.9300	C41—C42	1.393 (2)
C9—H9A	0.9300	C41—H41	0.9300
C9—H9B	0.9300	C43—C44	1.498 (3)
C10—C11	1.527 (2)	C43—H43A	0.9700
C11—C12	1.540 (2)	C43—H43B	0.9700
C11—H11	0.9800	C44—C45	1.313 (3)
C12—C13	1.518 (2)	C44—H44	0.9300
C12—H12	0.9800	C45—H45A	0.9300
C13—C14	1.388 (2)	C45—H45B	0.9300
C13—C18	1.392 (2)	C46—C47	1.528 (2)
C14—C15	1.393 (2)	C47—C48	1.538 (2)
C14—H14	0.9300	C47—H47	0.9800
C15—C16	1.381 (3)	C48—C49	1.516 (2)
C15—H15	0.9300	C48—H48	0.9800
C16—C17	1.381 (3)	C49—C54	1.390 (2)
C16—H16	0.9300	C49—C50	1.384 (2)
C17—C18	1.387 (2)	C50—C51	1.394 (3)
C17—H17	0.9300	C50—H50	0.9300
C18—H18	0.9300	C51—C52	1.372 (3)
C19—C20	1.397 (2)	C51—H51	0.9300
C19—C24	1.407 (2)	C52—C53	1.380 (3)
C20—C21	1.383 (3)	C52—H52	0.9300
C20—H20	0.9300	C53—C54	1.386 (3)
C21—C22	1.383 (3)	C53—H53	0.9300
C21—H21	0.9300	C54—H54	0.9300
C22—C23	1.382 (3)		
			
C11—O1—H1O	109.5	N4—C25—H25D	109.3
C29—O3—H3O	109.5	C26'—C25—H25D	109.3
C47—O5—H5O	109.5	H25C—C25—H25D	107.9
C1—N1—C12	121.97 (12)	C27—C26—C25	113.2 (4)
C1—N1—H1N	119.0	C27—C26—H26	123.4
C12—N1—H1N	119.0	C25—C26—H26	123.4
C10—N2—C6	122.30 (13)	C26—C27—H27A	120.0
C10—N2—C7	118.52 (13)	C26—C27—H27B	120.0
C6—N2—C7	118.62 (12)	H27A—C27—H27B	120.0
C19—N3—C30	120.48 (13)	C27'—C26'—C25	119.3 (4)
C19—N3—H3N	119.8	C27'—C26'—H26'	120.3
C30—N3—H3N	119.8	C25—C26'—H26'	120.3
C28—N4—C24	122.98 (14)	C26'—C27'—H27C	120.0
C28—N4—C25	117.62 (19)	C26'—C27'—H27D	120.0
C24—N4—C25	119.13 (19)	H27C—C27'—H27D	120.0
C37—N5—C48	117.89 (13)	O4—C28—N4	122.12 (19)
C37—N5—H5N	121.1	O4—C28—C29	118.8 (2)
C48—N5—H5N	121.1	N4—C28—C29	119.06 (15)
C46—N6—C42	122.04 (13)	O3—C29—C28	110.18 (14)
C46—N6—C43	118.52 (13)	O3—C29—C30	110.10 (14)
C42—N6—C43	119.05 (13)	C28—C29—C30	109.48 (14)
N1—C1—C2	120.46 (14)	O3—C29—H29	109.0
N1—C1—C6	121.63 (14)	C28—C29—H29	109.0
C2—C1—C6	117.72 (14)	C30—C29—H29	109.0
C3—C2—C1	121.18 (16)	N3—C30—C31	112.53 (13)
C3—C2—H2	119.4	N3—C30—C29	109.85 (13)
C1—C2—H2	119.4	C31—C30—C29	112.41 (13)
C2—C3—C4	120.56 (16)	N3—C30—H30	107.2
C2—C3—H3	119.7	C31—C30—H30	107.2
C4—C3—H3	119.7	C29—C30—H30	107.2
C5—C4—C3	119.28 (15)	C32—C31—C36	118.48 (16)
C5—C4—H4	120.4	C32—C31—C30	120.41 (14)
C3—C4—H4	120.4	C36—C31—C30	121.04 (15)
C4—C5—C6	120.40 (16)	C31—C32—C33	120.61 (16)
C4—C5—H5	119.8	C31—C32—H32	119.7
C6—C5—H5	119.8	C33—C32—H32	119.7
C5—C6—C1	120.53 (14)	C34—C33—C32	120.35 (16)
C5—C6—N2	118.90 (14)	C34—C33—H33	119.8
C1—C6—N2	120.50 (13)	C32—C33—H33	119.8
N2—C7—C8	114.44 (13)	C33—C34—C35	119.52 (17)
N2—C7—H7A	108.7	C33—C34—H34	120.2
C8—C7—H7A	108.7	C35—C34—H34	120.2
N2—C7—H7B	108.7	C36—C35—C34	120.17 (16)
C8—C7—H7B	108.7	C36—C35—H35	119.9
H7A—C7—H7B	107.6	C34—C35—H35	119.9
C9—C8—C7	127.04 (16)	C35—C36—C31	120.86 (16)
C9—C8—H8	116.5	C35—C36—H36	119.6
C7—C8—H8	116.5	C31—C36—H36	119.6
C8—C9—H9A	120.0	C38—C37—C42	118.93 (15)
C8—C9—H9B	120.0	C38—C37—N5	121.51 (15)
H9A—C9—H9B	120.0	C42—C37—N5	119.47 (14)
O2—C10—N2	122.25 (14)	C39—C38—C37	120.66 (17)
O2—C10—C11	121.09 (13)	C39—C38—H38	119.7
N2—C10—C11	116.67 (13)	C37—C38—H38	119.7
O1—C11—C10	110.52 (12)	C38—C39—C40	120.19 (17)
O1—C11—C12	111.50 (12)	C38—C39—H39	119.9
C10—C11—C12	108.47 (12)	C40—C39—H39	119.9
O1—C11—H11	108.8	C41—C40—C39	119.99 (17)
C10—C11—H11	108.8	C41—C40—H40	120.0
C12—C11—H11	108.8	C39—C40—H40	120.0
N1—C12—C13	112.64 (13)	C40—C41—C42	120.38 (17)
N1—C12—C11	110.39 (12)	C40—C41—H41	119.8
C13—C12—C11	111.80 (12)	C42—C41—H41	119.8
N1—C12—H12	107.2	C41—C42—C37	119.84 (15)
C13—C12—H12	107.2	C41—C42—N6	119.50 (15)
C11—C12—H12	107.2	C37—C42—N6	120.62 (14)
C14—C13—C18	118.85 (15)	N6—C43—C44	113.71 (14)
C14—C13—C12	119.57 (14)	N6—C43—H43A	108.8
C18—C13—C12	121.56 (14)	C44—C43—H43A	108.8
C13—C14—C15	120.59 (16)	N6—C43—H43B	108.8
C13—C14—H14	119.7	C44—C43—H43B	108.8
C15—C14—H14	119.7	H43A—C43—H43B	107.7
C16—C15—C14	120.07 (17)	C45—C44—C43	125.67 (16)
C16—C15—H15	120.0	C45—C44—H44	117.2
C14—C15—H15	120.0	C43—C44—H44	117.2
C15—C16—C17	119.66 (16)	C44—C45—H45A	120.0
C15—C16—H16	120.2	C44—C45—H45B	120.0
C17—C16—H16	120.2	H45A—C45—H45B	120.0
C16—C17—C18	120.46 (17)	O6—C46—N6	122.86 (15)
C16—C17—H17	119.8	O6—C46—C47	119.87 (14)
C18—C17—H17	119.8	N6—C46—C47	117.23 (13)
C17—C18—C13	120.36 (16)	O5—C47—C46	110.38 (13)
C17—C18—H18	119.8	O5—C47—C48	109.73 (13)
C13—C18—H18	119.8	C46—C47—C48	110.70 (13)
C20—C19—N3	120.85 (15)	O5—C47—H47	108.7
C20—C19—C24	118.27 (16)	C46—C47—H47	108.7
N3—C19—C24	120.58 (15)	C48—C47—H47	108.7
C21—C20—C19	121.01 (18)	N5—C48—C49	111.95 (13)
C21—C20—H20	119.5	N5—C48—C47	109.90 (12)
C19—C20—H20	119.5	C49—C48—C47	112.91 (13)
C20—C21—C22	120.01 (19)	N5—C48—H48	107.3
C20—C21—H21	120.0	C49—C48—H48	107.3
C22—C21—H21	120.0	C47—C48—H48	107.3
C23—C22—C21	120.05 (18)	C54—C49—C50	118.10 (16)
C23—C22—H22	120.0	C54—C49—C48	121.68 (14)
C21—C22—H22	120.0	C50—C49—C48	120.06 (15)
C22—C23—C24	120.43 (18)	C49—C50—C51	121.01 (18)
C22—C23—H23	119.8	C49—C50—H50	119.5
C24—C23—H23	119.8	C51—C50—H50	119.5
C23—C24—C19	120.22 (18)	C52—C51—C50	120.03 (18)
C23—C24—N4	119.95 (16)	C52—C51—H51	120.0
C19—C24—N4	119.64 (16)	C50—C51—H51	120.0
N4—C25—C26	112.3 (2)	C51—C52—C53	119.77 (17)
N4—C25—C26'	111.8 (2)	C51—C52—H52	120.1
N4—C25—H25A	109.1	C53—C52—H52	120.1
C26—C25—H25A	109.1	C52—C53—C54	120.12 (18)
N4—C25—H25B	109.1	C52—C53—H53	119.9
C26—C25—H25B	109.1	C54—C53—H53	119.9
H25A—C25—H25B	107.9	C49—C54—C53	120.97 (17)
N4—C25—H25C	109.3	C49—C54—H54	119.5
C26'—C25—H25C	109.3	C53—C54—H54	119.5
			
C12—N1—C1—C2	123.84 (16)	C25—N4—C28—O4	2.1 (3)
C12—N1—C1—C6	−61.3 (2)	C24—N4—C28—C29	9.3 (2)
N1—C1—C2—C3	177.18 (15)	C25—N4—C28—C29	−176.74 (15)
C6—C1—C2—C3	2.1 (2)	O4—C28—C29—O3	−19.3 (2)
C1—C2—C3—C4	2.6 (3)	N4—C28—C29—O3	159.57 (15)
C2—C3—C4—C5	−3.2 (3)	O4—C28—C29—C30	101.92 (18)
C3—C4—C5—C6	−0.9 (2)	N4—C28—C29—C30	−79.21 (19)
C4—C5—C6—C1	5.7 (2)	C19—N3—C30—C31	−89.64 (17)
C4—C5—C6—N2	−171.11 (14)	C19—N3—C30—C29	36.41 (19)
N1—C1—C6—C5	178.82 (14)	O3—C29—C30—N3	170.34 (13)
C2—C1—C6—C5	−6.2 (2)	C28—C29—C30—N3	49.08 (17)
N1—C1—C6—N2	−4.5 (2)	O3—C29—C30—C31	−63.54 (18)
C2—C1—C6—N2	170.56 (14)	C28—C29—C30—C31	175.20 (14)
C10—N2—C6—C5	−137.42 (15)	N3—C30—C31—C32	−119.09 (17)
C7—N2—C6—C5	51.28 (19)	C29—C30—C31—C32	116.26 (17)
C10—N2—C6—C1	45.8 (2)	N3—C30—C31—C36	57.7 (2)
C7—N2—C6—C1	−125.49 (15)	C29—C30—C31—C36	−66.9 (2)
C10—N2—C7—C8	−87.20 (17)	C36—C31—C32—C33	−0.9 (3)
C6—N2—C7—C8	84.43 (17)	C30—C31—C32—C33	176.02 (16)
N2—C7—C8—C9	4.8 (3)	C31—C32—C33—C34	−0.2 (3)
C6—N2—C10—O2	−170.89 (14)	C32—C33—C34—C35	0.8 (3)
C7—N2—C10—O2	0.4 (2)	C33—C34—C35—C36	−0.4 (3)
C6—N2—C10—C11	8.9 (2)	C34—C35—C36—C31	−0.6 (3)
C7—N2—C10—C11	−179.78 (12)	C32—C31—C36—C35	1.3 (3)
O2—C10—C11—O1	−24.25 (19)	C30—C31—C36—C35	−175.59 (17)
N2—C10—C11—O1	155.95 (13)	C48—N5—C37—C38	−111.46 (17)
O2—C10—C11—C12	98.27 (16)	C48—N5—C37—C42	71.98 (19)
N2—C10—C11—C12	−81.53 (15)	C42—C37—C38—C39	0.1 (3)
C1—N1—C12—C13	−96.21 (16)	N5—C37—C38—C39	−176.51 (16)
C1—N1—C12—C11	29.55 (19)	C37—C38—C39—C40	−0.8 (3)
O1—C11—C12—N1	176.81 (12)	C38—C39—C40—C41	0.3 (3)
C10—C11—C12—N1	54.89 (16)	C39—C40—C41—C42	0.8 (3)
O1—C11—C12—C13	−56.96 (17)	C40—C41—C42—C37	−1.5 (3)
C10—C11—C12—C13	−178.88 (12)	C40—C41—C42—N6	176.56 (16)
N1—C12—C13—C14	−128.67 (16)	C38—C37—C42—C41	1.1 (2)
C11—C12—C13—C14	106.34 (17)	N5—C37—C42—C41	177.70 (15)
N1—C12—C13—C18	49.6 (2)	C38—C37—C42—N6	−176.99 (15)
C11—C12—C13—C18	−75.35 (19)	N5—C37—C42—N6	−0.3 (2)
C18—C13—C14—C15	−1.0 (3)	C46—N6—C42—C41	137.53 (16)
C12—C13—C14—C15	177.31 (16)	C43—N6—C42—C41	−49.8 (2)
C13—C14—C15—C16	0.2 (3)	C46—N6—C42—C37	−44.4 (2)
C14—C15—C16—C17	0.9 (3)	C43—N6—C42—C37	128.29 (16)
C15—C16—C17—C18	−1.2 (3)	C46—N6—C43—C44	95.05 (18)
C16—C17—C18—C13	0.4 (3)	C42—N6—C43—C44	−77.92 (19)
C14—C13—C18—C17	0.7 (3)	N6—C43—C44—C45	−6.1 (3)
C12—C13—C18—C17	−177.58 (16)	C42—N6—C46—O6	174.05 (15)
C30—N3—C19—C20	116.39 (17)	C43—N6—C46—O6	1.3 (2)
C30—N3—C19—C24	−70.0 (2)	C42—N6—C46—C47	−8.5 (2)
N3—C19—C20—C21	172.62 (16)	C43—N6—C46—C47	178.74 (13)
C24—C19—C20—C21	−1.1 (2)	O6—C46—C47—O5	18.0 (2)
C19—C20—C21—C22	0.0 (3)	N6—C46—C47—O5	−159.56 (14)
C20—C21—C22—C23	1.0 (3)	O6—C46—C47—C48	−103.73 (17)
C21—C22—C23—C24	−0.9 (3)	N6—C46—C47—C48	78.75 (17)
C22—C23—C24—C19	−0.3 (3)	C37—N5—C48—C49	85.17 (17)
C22—C23—C24—N4	−175.14 (17)	C37—N5—C48—C47	−41.14 (18)
C20—C19—C24—C23	1.2 (2)	O5—C47—C48—N5	−168.89 (13)
N3—C19—C24—C23	−172.53 (15)	C46—C47—C48—N5	−46.82 (17)
C20—C19—C24—N4	176.14 (15)	O5—C47—C48—C49	65.34 (17)
N3—C19—C24—N4	2.4 (2)	C46—C47—C48—C49	−172.59 (13)
C28—N4—C24—C23	−143.55 (17)	N5—C48—C49—C54	−68.0 (2)
C25—N4—C24—C23	42.6 (2)	C47—C48—C49—C54	56.6 (2)
C28—N4—C24—C19	41.5 (2)	N5—C48—C49—C50	107.31 (17)
C25—N4—C24—C19	−132.32 (17)	C47—C48—C49—C50	−128.03 (16)
C28—N4—C25—C26	64.3 (3)	C54—C49—C50—C51	−0.8 (3)
C24—N4—C25—C26	−121.5 (3)	C48—C49—C50—C51	−176.27 (16)
C28—N4—C25—C26'	91.2 (3)	C49—C50—C51—C52	0.2 (3)
C24—N4—C25—C26'	−94.7 (3)	C50—C51—C52—C53	0.2 (3)
N4—C25—C26—C27	130.7 (4)	C51—C52—C53—C54	−0.1 (3)
C26'—C25—C26—C27	36.7 (6)	C50—C49—C54—C53	0.9 (3)
N4—C25—C26'—C27'	−131.1 (4)	C48—C49—C54—C53	176.30 (17)
C26—C25—C26'—C27'	−34.9 (6)	C52—C53—C54—C49	−0.4 (3)
C24—N4—C28—O4	−171.86 (16)		

### Hydrogen-bond geometry (Å, °)

**Table:**

*D*—H···*A*	*D*—H	H···*A*	*D*···*A*	*D*—H···*A*
O1—H1o···O2^i^	0.84	1.96	2.786 (2)	167
O5—H5o···O6^ii^	0.84	2.30	3.022 (2)	145
N1—H1n···O3	0.88	2.58	3.142 (2)	123
N3—H3n···O4^iii^	0.88	2.40	2.900 (2)	116

Symmetry codes: (i) −*x*+1, *y*, −*z*+1/2; (ii) −*x*+1/2, −*y*+3/2, −*z*+1; (iii) *x*, −*y*, *z*−1/2.
